# Investigating mental health service user views of stigma on Twitter during COVID-19: a mixed-methods study

**DOI:** 10.1080/09638237.2022.2091763

**Published:** 2022-07-03

**Authors:** Sonja M. Jansli, Georgie Hudson, Esther Negbenose, Sinan Erturk, Til Wykes, Sagar Jilka

**Affiliations:** a Institute of Psychiatry, Psychology and Neuroscience, King’s College London, London, UK; b South London and Maudsley NHS Foundation Trust, London, UK; c Warwick Medical School, University of Warwick, Coventry, UK

**Keywords:** patient and public involvement, mental health, co-production, stigma, Twitter, social media, England

## Abstract

**Background:** Mental health stigma on social media is well studied, but not from the perspective of mental health service users. Coronavirus disease-19 (COVID-19) increased mental health discussions and may have impacted stigma.

**Objectives:** (1) to understand how service users perceive and define mental health stigma on social media; (2) how COVID-19 shaped mental health conversations and social media use.

**Methods:** We collected 2,700 tweets related to seven mental health conditions: schizophrenia, depression, anxiety, autism, eating disorders, OCD, and addiction. Twenty-seven service users rated them as stigmatising or neutral, followed by focus group discussions. Focus group transcripts were thematically analysed.

**Results:** Participants rated 1,101 tweets (40.8%) as stigmatising. Tweets related to schizophrenia were most frequently classed as stigmatising (411/534, 77%). Tweets related to depression or anxiety were least stigmatising (139/634, 21.9%). A stigmatising tweet depended on perceived intention and context but some words (e.g. “psycho”) felt stigmatising irrespective of context.

**Discussion:** The anonymity of social media seemingly increased stigma, but COVID-19 lockdowns improved mental health literacy. This is the first study to qualitatively investigate service users' views of stigma towards various mental health conditions on Twitter and we show stigma is common, particularly towards schizophrenia. Service user involvement is vital when designing solutions to stigma.

## Introduction

Despite the high prevalence of mental health conditions, mental health stigma is common, leading to negative health outcomes (Budhwani & De, 2019; Karamouzian et al., 2018; Pachankis et al., 2018) and making help seeking less likely (Gulliver et al., 2012; Yap et al., 2013). Stigma is “an attribute that is deeply discrediting” which reduces someone “from a whole and usual person to a tainted, discounted one” (Goffman, 1963, p. 3). But how people define and experience stigma is deeply personal, relying on factors including context and power dynamics. Mental health is frequently discussed on Twitter and can provide community, support, and information for people to manage their mental health (Berry et al., 2017). But it also allows the propagation of stigmatising attitudes, which can become internalised, leading to distrust of health professionals, scepticism of public health systems, and an unwillingness to disclose behaviours related to mental health (Budhwani & De, 2019; Turan et al., 2017).

Robinson et al. (2019) analysed tweets related to both physical and mental health, finding that mental health conditions are stigmatised more than physical health on social media (12.9% versus 8.1%, respectively). Budenz et al. (2020) also investigated stigma towards mental health conditions on Twitter, finding low levels of stigma towards mental illness generally (2.6%) but much higher levels towards bipolar disorder. However, bipolar disorder was the only mental illness specifically investigated. They were unable to compare the prevalence of stigma towards other conditions. Moreover, in both studies, researchers decided whether the tweet reflected stigma, rather than people with experience of mental health problems. Research is limited from service users’ perspectives. Green et al. (2003) investigated how stigma affects service users, by involving them in the research methodology, and found stigma had a large impact on those who have used mental health services. However, they did not ask participants how they *defined* stigma and their conclusions are limited by a homogeneous sample. To the best of our knowledge, no other studies have asked mental health service users how they experience and define stigma on Twitter.

The Coronavirus disease-19 (COVID-19) pandemic negatively affected mental health and wellbeing (Vindegaard & Benros, 2020), which may have influenced how individuals discuss mental health on Twitter. In the UK, people experienced increased insomnia, anxiety, low mood, and general psychological distress during the pandemic and its “lockdowns” (Niedzwiedz et al., 2021; Pieh et al., 2021; Pierce et al., 2020). As increased awareness of mental health reduces stigma towards it (Spagnolo et al., 2008), we wanted to understand service user experiences of mental health stigma on social media during the pandemic. Involvement through participatory methods (Rose, 2018; Wykes, 2014) is a vital first step towards understanding what service users consider to be stigma online. We overcome this gap by co-developing with service users a framework of mental health stigma on Twitter. We aim to investigate how those with experiences of mental health problems perceive and define mental health stigma on social media, and how the COVID-19 pandemic has shaped social media usage and content.

## Methods

### Design

This was a cross-sectional mixed-methods study using semi-structured focus groups with mental health service users to investigate: (1) how service users perceive and define mental health stigma on social media; and (2) how COVID-19 has shaped mental health conversations and social media use. This study was granted ethical approval from East Midlands - Derby Research Ethics Committee on 25th November 2020 (ref: 20/EM/0274).

### Patient involvement

Mental health service users were involved as participants. Service user researchers (researchers with experience of using mental health services) were key in the design, project management and data analysis.

### Participants and recruitment

Participants were recruited via mental health research advisory groups (e.g. the Maudsley Biomedical Research Centre’s advisory groups; NIHR Maudsley Biomedical Research Centre, 2021). Eligibility criteria included: current or previous UK mental health service use, aged 18 or over, ability to give informed consent and communicate in English, and access to the internet and a suitable device for the focus groups. All participants consented to their data being used in the research.

## Procedure

### Tweet Extraction

The research team extracted 72,731 tweets using Twitter’s Application Programming Interface (API; Twitter, 2021) via the Tweepy Python library (Tweepy, 2021). To extract these tweets, the team used keywords relating to seven of the most stigmatised conditions: Schizophrenia, Depression, Anxiety, Autism, Eating Disorders, obsessive compulsive disorder (OCD) (Robinson et al., 2019) and Addiction (Matthews et al., 2017). Search terms were based on those used previously in similar work (Robinson et al., 2019; Jilka et al., 2022; see supplementary material for keywords). We collected tweets during UK office hours (9 am–5 pm) spanning various pandemic stages: pre-UK lockdown (1st January–22nd March 2020), the first period of lockdown (23rd March–30th April 2020), and changing of lockdown rules (1st May–31st December 2020). There were no restrictions on user locations or user-types. From these extracted tweets, we randomly selected 2,700 tweets to provide 100 for each of the 27 participants. We sought to make this sample an even representation of the seven mental health conditions and each period of change in policy regarding lockdown and the COVID-19 pandemic. We followed the Community Principles on Ethical Data Sharing (CPEDS; Green, 2022). As publicly available information, tweets provided to participants were original, with no paraphrasing to ensure the meanings and intentions in the tweets were conserved. However identifiable information including usernames were removed from the tweets before they were given to participants.

### Tweet rating

**Figure 1.**
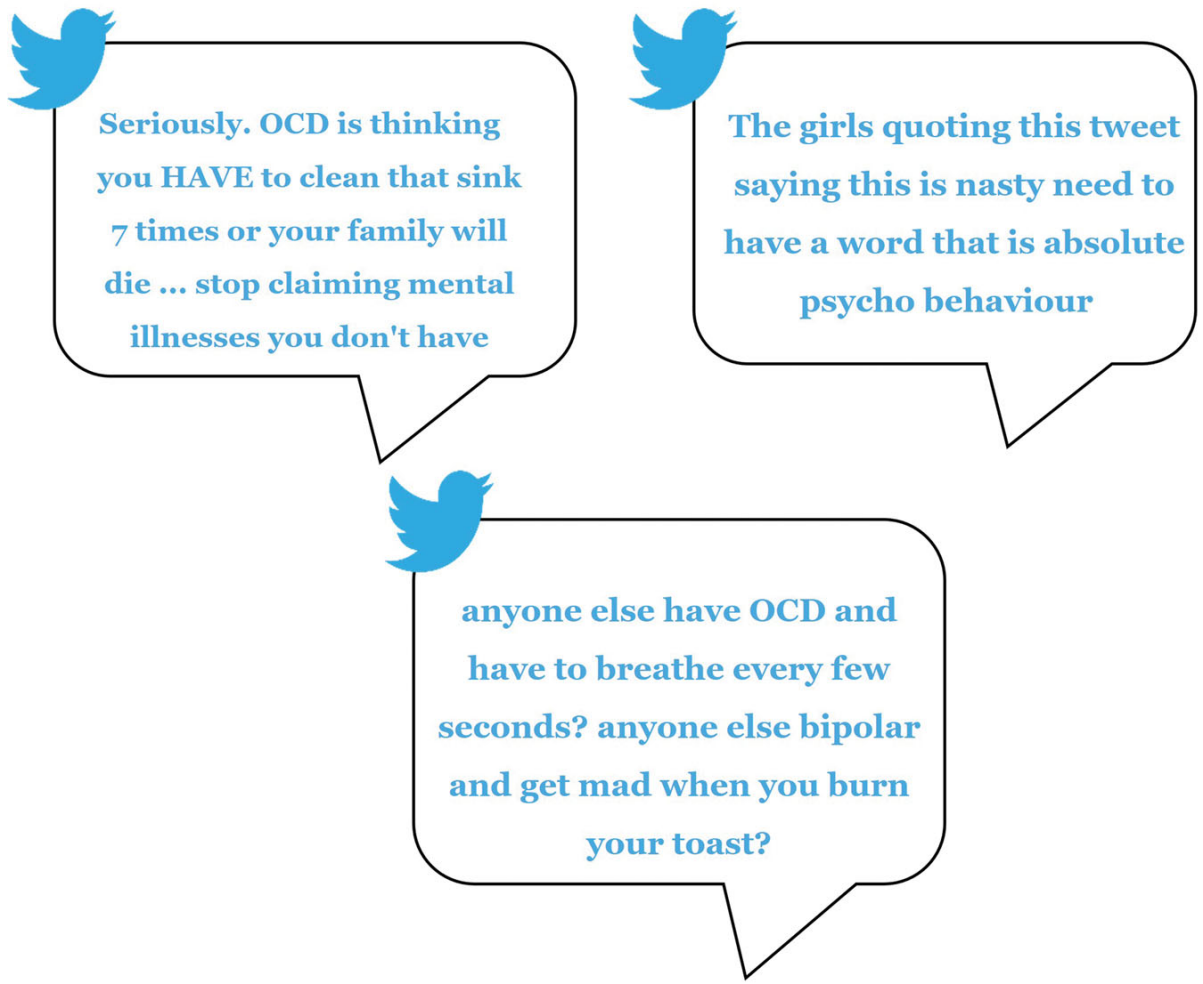
Example tweets for participant rating.

After completing a demographics questionnaire, participants were given 100 mental health related tweets to review and classify as stigmatising or neutral to ensure a shared understanding of tweet content. Each participant’s set of tweets contained tweets from the seven most stigmatised conditions. See figure 1 for three example tweets extracted for participants to rate.

### Focus groups

Participants were then invited to take part in an online focus group to discuss their reasons for classifying a tweet as stigmatising, and their experiences of using social media during the COVID-19 pandemic, and its effects on their mental health. See supplementary material for the topic guide. Four focus groups of 5–8 participants took place, each scheduled to last 90 min. They were facilitated by service user researchers approximately one month after tweet-rating.

### Thematic analysis and member checking

After each focus group, service user researchers conducted an initial thematic analysis and invited participants to take part in one of four online member-checking groups to assess theme accuracy, allowing participants to expand on their views. All focus groups complied with COVID-19 regulations and were held virtually via Microsoft Teams between February and April 2021.

### Inter-rater reliability

Inter-rater reliability was assessed by 10 of the service users classifying a further 100 tweets that had previously been rated as either stigmatising or neutral. A total of 1,000 tweets were used for the inter-rater reliability assessment.

## Data analysis

An inter-rater reliability analysis using the Kappa statistic was performed to determine consistency among service users. We report the average Kappa score and the average percentage agreement.

Differences in the rates of stigma between the mental health conditions were assessed using the chi-square test.

Researchers thematically analysed the focus group transcripts using Braun and Clarke’s (2006) six phases of thematic analysis. Three service user researchers independently coded each transcript using NVivo 12, resulting in a thematic framework for each focus group. They then created deductive codes using the topic guide prompts, leading to inductive codes. Themes were amended if necessary, after the member-checking exercise.

The service user researchers created the final thematic framework together, discussing similarities and differences across each framework, and using elements of the multiple coding approach (Sweeney et al., 2013). Researchers then reviewed the themes for research question relevance and to ensure they were coherent and distinctive. Themes deemed discrete or not supported with enough data were discarded (Braun & Clarke, 2006). Data collection continued until data saturation at the theme level was reached, which was determined by a review of the summary findings from each focus group (Guest et al., 2017). These analyses led to the creation of a final thematic framework, reflecting common themes across all four focus groups.

## Results

### Participant characteristics

Participant characteristics of the 27 service users who rated 2,700 tweets are summarised in Table 1.

**Table 1.** Participant characteristics (*N* = 27).

Characteristic	
Gender, n (%)	
Female	21 (77.8%)
Male	4 (14.8%)
Other	2 (7.4%)
Gender assigned at birth, *n* (%)	
Female	22 (81.5%)
Age (years), mean (SD)^a^	45.23 (14.88)
	Range: 19–69
Ethnicity, *n* (%)	
Asian/Asian British	3 (11.1%)
Black/African/Caribbean/Black British	2 (7.4%)
Mixed/Multiple ethnic groups	1 (3.7%)
White	21 (77.8%)
Degree level qualification or above, *n* (%)	19 (70.4%)
Age completed studies (years), mean (SD)^a^	22.54 (6.11)
Currently receiving support for mental health, *n* (%)	19 (70.4%)

*Note*. ^a^*n* = 1 missing data.

### Service user ratings of tweets

Participants rated 1,101 tweets (40.8%) as stigmatising. There were statistical differences in the proportion of tweets service users rated as stigmatising, depending on the mental health condition they referred to (*χ*^2^(5) = 474.48, *p* < .001). Tweets related to schizophrenia were most commonly classed as stigmatising (411/534, 77.0%) and tweets related to depression or anxiety were least frequently classed as stigmatising (139/634, 21.9%). See Supplementary Table 1 for a breakdown by condition.

There was fair agreement between service users on average (*κ* = 0.4; average agreement 71.00%) when service users rated tweets as stigmatising or neutral.

### Focus group themes

Twenty-three participants took part in the focus groups (see Supplementary Table 2 for details). All but two focus group participants took part in a member-checking group.

We generated 16 themes within a framework of four categories, which are presented in Table 2.

**Table 2.** Framework categories, themes, and subthemes, showing the focus group(s) each subtheme appeared in.

Framework category	Themes	Subthemes	FG1	FG2	FG3	FG4
Defining stigma and stigmatising language	Intention and Context	Misuse of official mental health terminology	✓	✓	✓	✓
		Self-labelling	✓		✓	✓
		Psycho	✓	✓	✓	✓
		Everyday language	✓	✓	✓	
	Intuition and Feeling	Considering how others may feel	✓		✓	✓
		Personal connection		✓	✓	✓
Social media culture and how it shapes mental health	Depiction of mental health		✓		✓	✓
	Anonymity on social media		✓	✓		✓
	Content management issues				✓	✓
	Vulnerability of children and people with mental illnesses		✓	✓	✓	✓
	Negative effects of too much social media use			✓	✓	
	Self-portrayal on social media and Comparisons			✓		
	Source of support and community			✓	✓	✓
The effect of COVID-19 on social media use/content and mental health	Changes in content	Early versus late stage of the pandemic		✓	✓	✓
		Divisiveness		✓	✓	✓
		Mental health is discussed more	✓		✓	
		Helpful and supportive content	✓	✓	✓	✓
	Changes in use	Increase in social media use	✓	✓	✓	✓
		Selective use				✓
		Differences in platforms			✓	✓
		No changes in content or use		✓	✓	✓
Solutions to combat social media stigma	People	Cross-sectional conversations	✓	✓	✓	✓
		Educating children	✓			
	Social media	Investing in admins, moderators, and other support			✓	✓
		Warning and account suspension				✓
		Improving protection from triggers	✓	✓	✓	✓
		Awareness campaigns				✓

## Defining stigma and stigmatising language

### Intention and context

If it appeared **official mental health terminology** was being used derogatorily or inappropriately diagnostically, this was generally seen as stigmatising, as it “*de-emphasises or belittles the importance about how effective that word can be”* (Participant 29, Group 4). It was felt to be less stigmatising if the person was **self-labelling**, for example “*somebody who had experience who was trying to use it as a way of coping with the world”* (Participant 3, Group 1). Others felt that the use of stigmatising words may be triggering regardless. For instance, the word “**psycho**” was classified as stigmatising by almost all participants, regardless of context, but tweets containing other mental health related words were felt to be *“not quite as punishing […] as some of those […] psycho references”* (Participant 10, Group 1*).* Participants believed the word “psycho” referred to any mental health condition*, “essentially anyone who doesn't act like the norm”* (Participant 7, Group 1), not just schizophrenia. Participants acknowledged many words have become normalised and are used in **everyday language,** without the person realising or intending to cause harm.

### Intuition and feeling

Participants used their intuition when rating tweets, which included **considering how others may feel** reading the tweet, “*irrespective of whether [they themselves] found it offensive or not”* (Participant 10, Group 1). Participants found tweets to be triggering “*particularly if you are struggling with […] a condition which is being trivialised”* (Participant 23, Group 2). This **personal connection** to the condition made participants more likely to rate it as stigmatising.

## Social media culture and how it shapes mental health

Participants felt the **depiction of mental health** online was often negative, with mental health conditions ridiculed or *“categorised as being sometimes a horrific thing”* (Participant 8, Group 1). This culture was thought to be particularly negative on Twitter because Twitter offers **anonymity,** where it “*is easier to hide […] whereas if you look at Facebook or Instagram, there's stuff that makes it more personal […] and arguably therefore people are a bit nastier on it [Twitter]”* (Participant 29, Group 4). It is this anonymity on Twitter which participants thought lead to this negative depiction of mental health on Twitter, as “a *lot of people […] seem to feel they can say things that they wouldn't say to people face to face”* (Participant 8, Group 1). However, there appeared to be differences in the way mental health conditions were discussed on Twitter, with *“some lesser understood or lesser-known mental health conditions, […] like schizophrenia or psychosis or personality disorder”* (Participant 28, Group 4) portrayed more negatively and receiving less support during events such as Mental Health Awareness week.

Participants also criticised the ways in which social media sites **managed their content** and felt that sites like Twitter did not protect users enough from triggering content, allowing users to “*pretty much say what they want, and it takes a long time before […] they might suspend someone's account and stop them posting certain language”* (Participant 22, Group 4). Some participants also highlighted the **vulnerability of children and people with mental illnesses** on social media, who may be more affected by triggering content or even post on social media *“without really realising the implications of it, without really realising that it might not be appropriate, or it might not be in their best interest to do that”* (Participant 9, Group 3). Many participants noted **negative effects of too much social media use**, such as wasting time, reducing one’s attention span, losing touch with real life, and getting drawn into debates. In addition, users’ **self-portrayal on social media** was felt to be often highly exaggerated and **comparing** oneself to others online could make people *“feel quite unsuccessful the whole time”* (Participant 7, Group 2). The fictitious nature of social media led the participants to feel unhappy when comparing themselves to others on social media as “*I've been like, oh God and this person [is] selling loads of stuff […] and I found out that actually they haven't sold anything*” (Participant 23, Group 3). This opinion was seen to be particularly prevalent on Instagram as “*there's a lot of filters on there and people can make themselves look more glammed up*” (Participant 14, Group 3), which can “*make you feel really inferior and useless*” (Participant 23, Group 3). Nevertheless, social media was also seen to be a **source of support and community**, where people could access useful information, reach out for help, and raise awareness.

## The effect of COVID-19 on social media use/content and mental health

Many participants described how the pandemic had adversely affected their mental health, due to isolation and boredom, as well as having increased difficulties accessing mental health services. Participants also noticed changes in social media usage and content resulting from the COVID-19 pandemic.

### Changes in content

Participants noticed clear differences in mood reflected on social media when comparing the **early and later stages of the pandemic**. Posts appeared to reflect more optimism and *"a sense of community spirit”* (Participant 9, Group 3) in the first UK lockdown (March 2020), but as the pandemic continued, “*good will […] dropped slightly [as] most people haven't really seen much change in the last year and […] the content […] is quite repetitive”* (Participant 29, Group 4). There also seemed to be more **divisiveness** on social media. While some felt this was in relation to “*social media in general, it's […] really quite a depressing place to be. There's lots of like triggering content or people like talking about things they don't really have an understanding of or just straight up offensive things*” (Participant 16, Group 3), some participants felt this divisiveness on Twitter became more extreme as a result of COVID-19 (“*I've used Twitter quite a lot like throughout the years pre-pandemic and currently […] I've noticed that even though I have a very well curated, lovely, happy Twitter timeline, that since the pandemic there's been a lot more divisiveness” (Participant 28, Group 4),.* Some participants felt this divisiveness intensified as they felt increasingly frustrated or angry at posts related to a lack of compliance with COVID-19 restrictions (“*there was a lot of people going against it [lockdown] stuff like that, so there was a lot of parties, and it was quite damaging to see as well.” Participant 4, Group 3)*. **Mental health was discussed more**, which appeared to be a result of the pandemic and the potential increase in mental health issues. This was felt to be positive, as people were now able to discuss their experiences more openly. However, these discussions could feel negative, in particular when situational mental health issues were being treated like serious mental illnesses and “*people are proud or bragging about the fact that […] they are experiencing some depression or anxiety and it's sort of downplaying what [people with serious mental illnesses] have been living with for years and […] putting it on the same level”* (Participant 7, Group 1).

On the other hand, participants noted that social media offered lots of **helpful and supportive content** throughout the pandemic and allowed people to connect to their loved ones and access useful resources.

### Changes in use

Overall participants felt their **social media use had increased** since the start of the pandemic as they had more free time, were sometimes lonely, and it provided distraction from the pandemic. Some tried to use social media **selectively**, for example feeling less need to keep up with COVID-19 related news, but also because it left them “*not always feeling much happier, [which is] difficult when you don't have a lot going on in a lockdown”* (Participant 29, Group 4). Participants changed the way they used **different platforms**. Twitter appeared to be more negative throughout the pandemic since it creates an environment for arguments and debates. Some participants preferred Instagram, TikTok and Nextdoor, to keep in contact with friends and family, find out important information, and for entertainment.

Some participants felt that content or usage had **not changed** as a result of the COVID-19 pandemic. It seemed that “*Twitter was the same as it was three years ago, before the pandemic”* (Participant 15, Group 3) and divisiveness and debates had always been present, but the topic of discussion had changed.

## Solutions to combat social media stigma

### People

Participants discussed **cross-sectional conversations** spanning different ages, cultural groups and amongst friends and family, as well as involving external organisations, to improve people’s understanding of mental illness and reduce misconceptions and stigma. It was felt that *“ending stigmatisation of mental health is not funded very well and it's not a priority for this government”* (Participant 26, Group 4) and talking to others about one’s own lived experiences could be the first steps toward reducing stigmatising beliefs. **Educating children** in schools and at home was seen as a way to prevent younger generations from developing stigmatising beliefs. Schools could implement initiatives in which people with lived experience give students an *“insider’s guide to mental health*” (Participant 3, Group 1) and teachers who hear students use stigmatising language should *“intervene in some way […] and try and educate rather than telling somebody off”* (Participant 2, Group 1).

### Social media

Participants felt that social media sites should **invest more in moderators** to monitor stigmatising and offensive language, as well as act as sources of support for users who become distressed through offensive posts and conversations. Those using offensive language should receive a **warning and subsequent account suspension** if the behaviour continues. Participants suggested companies should **improve mechanisms to protect them from triggers,** for example by blurring sensitive content and providing filters for certain words and phrases. Some participants also proposed taking *“a proactive approach as well as […] reactive”* (Participant 28, Group 4) via **awareness campaigns,** in particular if bigger corporations took an interest in advocating against mental health stigma.

## Discussion

We found a high level of perceived stigma among the 2,700 mental health-related tweets, with schizophrenia being the most stigmatised condition. Service users helped us improve existing definitions of stigma, such as the misuse of mental health terminology and particularly the word “psycho”. They reported mental health content on Twitter became more positive in the first stages of COVID-19, however this decreased over time and often was reserved for people suffering from temporary or less severe mental health problems.

Although mental health stigma on social media has been investigated previously (Budenz et al., 2020; Robinson et al., 2019), we focus solely on views of those with experience of mental health conditions, with service user researchers central to data collection and analysis. Additionally, this study is unique as service users, rather than researchers, rated whether the tweets were stigmatising, ensuring the definitions of stigma are rooted in their perspectives. Using this methodology, service users found stigma in three to 15 times more mental health related tweets (40.8%) than the 12.9% (Robinson et al., 2019) and 2.6% (Budenz et al., 2020) of tweets rated by researchers as stigmatising in prior research. Our findings corroborate Robinson et al.’s (2019) findings that schizophrenia is the most stigmatised mental health condition. We demonstrate that service users consider issues stigmatising which non-users do not. Therefore, future research should involve service users as their perceptions of stigma differ dramatically from researchers’.

**Figure 2.**
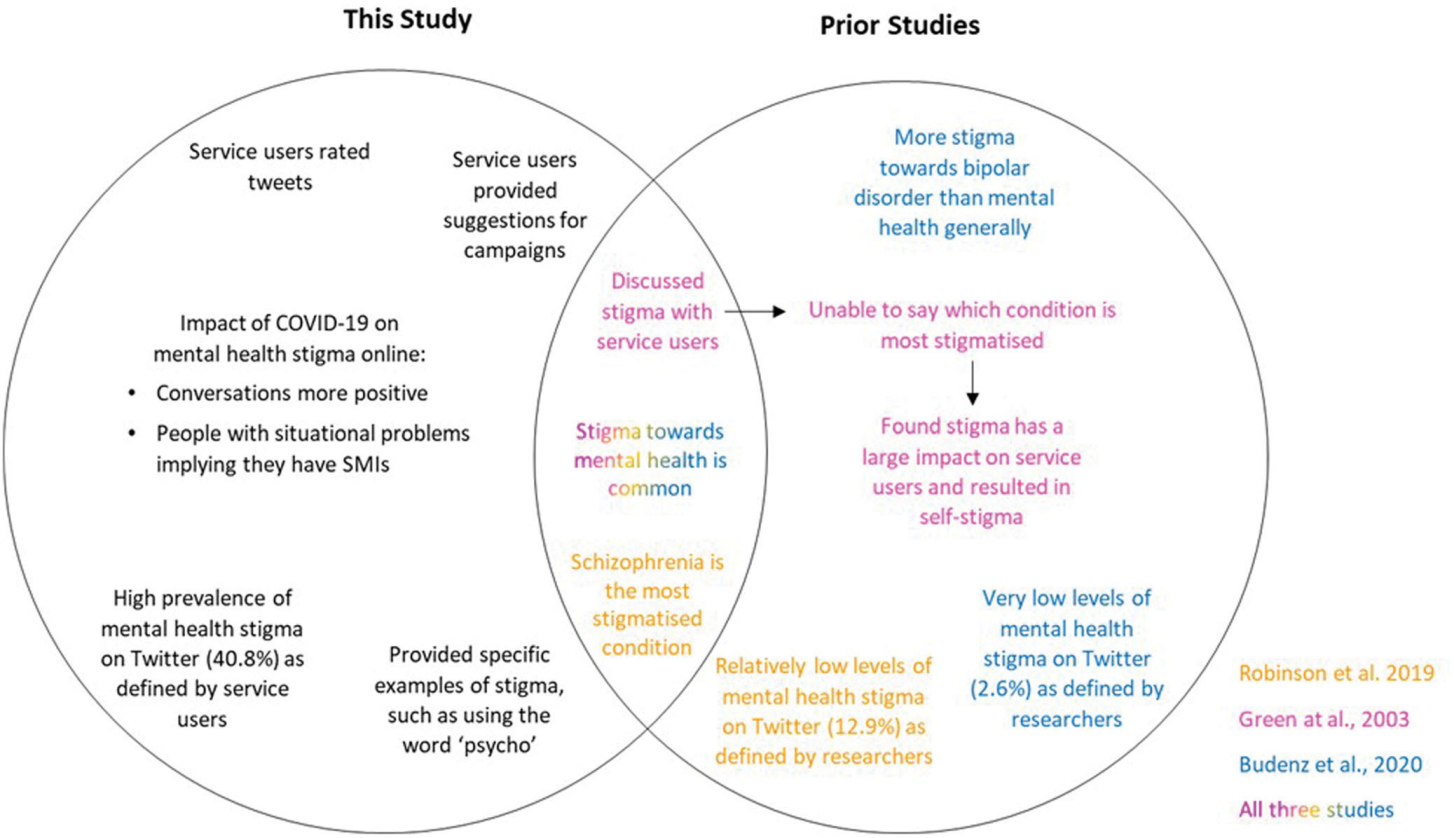
A Venn diagram comparing the current study with previous literature of mental health stigma.

Our high prevalence of stigma reflects findings by Green et al. (2003) who found all service users reported being affected by stigma and perceived high levels of stigma. We expand their work to understand which conditions service users find most stigmatised and how they define stigma. We highlight how our work differentiates from previous work in Figure 2.

### Service user views of online stigma and stigmatising language

Participants highlighted the difficulty in defining mental health stigma, as it is subjective and dependent on various factors. Nevertheless, they used intuition when rating the tweets and gave concrete examples where mental health stigma may occur. Tweets that appeared to misuse mental health terminology were considered stigmatising, mirroring previous research (Pavelko & Myrick, 2016). There was disagreement about the use of derogatory mental health terms, especially if seemingly used to cope with one’s own mental health issues. Self-labelling can help those with lived experience reclaim words otherwise used to describe mental illness in a derogatory way (Laverack, 2013), and therefore complete censorship of such words may be counterproductive. Importantly, participants in our study considered the word “psycho” stigmatising towards all mental health conditions, not just schizophrenia. Promoting awareness around the use of words which can perpetuate mental health stigma may therefore be beneficial for the reduction of stigma related to any mental illness (Wahl & Harman, 1989).

### Service user opinions of social media content and culture during COVID-19

Some participants found Twitter had a negative effect on their mental wellbeing during COVID-19, preferring other platforms such as Instagram and TikTok. This contrasts with research that demonstrated both passive and active Twitter use were positively related to life satisfaction and TikTok use did not impact wellbeing (Masciantonio et al., 2021). We focused specifically on the effects of social media on mental health service users, not the general population, and our findings highlight the importance of investigating the views of service users, who may be more vulnerable to the negative effects on mental health.

There is limited research investigating social media usage over the pandemic, but the increased use reported by our participants is consistent with reports from statistic-tracking companies. Forty-four percent of 12,845 people surveyed across 13 countries, and 21% of people in the UK, reported that they were spending longer on social media (Statistica, 2021). We did not quantitatively measure participants’ social media use, however, our participants described an increase in social media use during COVID-19, and their reflections during this period go some way to understanding mental health and social media use during the pandemic.

Service users reported changes in social media content throughout the pandemic. Initially, the general tone was supportive and positive, enabling people to connect and share resources. However, as the lockdowns continued, social media content became increasingly divisive and was used as a medium for people to vent frustrations. This could also be linked to social media usage; Zhong et al. (2021) found that social media usage was rewarding in COVID-19’s early stages, resulting in informational, emotional, and peer support, but excessive use was related to mental health issues. Service users also reported that mental health social media content became more positive and more frequently discussed during the first lockdown. However, these discussions seemed only related to situational mental health problems arising from the pandemic and were often conflated with serious mental health illnesses. Participants felt that those who continue to experience stigma due to their mental health condition were not included in the discussions around mental health awareness. Previous research has not investigated the content of social media mental health discussions during the COVID-19 pandemic. We were able to gain a high-level understanding of social media content relating to mental health and how this changed through the course of the COVID-19 pandemic.

### Solutions

Solutions included actions that can be taken by individuals, social media companies and other organisations. Having conversations across different cultural and age groups to improve the understanding of mental health was emphasised. Research on reducing stigma supports these conclusions (Corrigan et al., 2012; Evans-Lacko et al., 2010; Spagnolo et al., 2008) but none of this literature specifically relates to reductions of stigmatising attitudes or posts on social media. The approaches suggested by service users to minimise stigma corroborate the current evidence and are therefore likely to achieve some reductions in mental health stigma, but whether this can reduce stigma specifically on social media needs further investigation. Participants suggested solutions that can be actioned by social media companies, including warnings, account suspensions, investing more in moderators, and improving mechanisms to protect users from triggers. Although these suggestions are already used to some extent by social media platforms, participants still reported a high level of stigma on social media, and therefore social media companies should involve service users in the development of their apps. Any social media campaign must involve service users to ensure that they focus on aspects of stigma that are most important to them.

## Strengths and limitations

Although our sample was largely white British, we have improved upon previous work (Green et al., 2003) with a more heterogenous sample. Some sub-themes only appeared occasionally, but this does not mean these themes are less important (Pyett, 2003), as participants may simply have lacked opportunities to mention particular issues in some focus groups. However, further investigation may be needed to validate these sub-themes or capture new ones. We also cannot distinguish between user opinions at different lockdowns, as the focus groups discussed social media content as a whole. Future work should investigate how different policies at different timepoints affected public perceptions of mental health stigma.

Service user researchers were employed throughout this project and led on recruitment and data collection, which may have encouraged more self-disclosure by participants. It has been argued service user interviewers increase the comfort of research participants, improving the validity of results (Croft et al., 2016; Ostrow et al., 2017). Additionally, service user researchers analysed the data, providing a contextual understanding to the transcripts.

We did not measure whether the participants were social media or Twitter users, therefore their opinions may not be representative of other service users who regularly use Twitter. Future work should ensure all participants are Twitter-users and compare their views to the current study. Additionally, we did not assess participants’ mental health diagnoses. It is possible individual experiences of various mental health problems may impact their views of stigma; therefore future work should ensure a diverse range of mental health conditions in participants.

We carried out an inter-rater reliability analysis to understand how service users’ views of stigma differ and found fair/moderate agreement. This highlights the difficulty in capturing service user views of mental health stigma and the heterogeneity in mental health conditions, combined with societal and cultural factors which shape how individuals communicate and understand their mental health (Hudson et al., 2022; Taylor & Brown, 1988).

## Conclusions

To the best of our knowledge, stigma has not been defined with service users in the modern context of social media. Stigma is a complex phenomenon and service users defined stigma based on their intuition and experiences. We show mental health stigma is common on Twitter, particularly towards schizophrenia. Service users thought mental health was discussed more frequently during COVID-19, but this public awareness may diminish the experiences of individuals with serious mental illness. Some words (e.g. “psycho”) were universally regarded as stigmatising by service users. They suggested solutions to online stigma, such as education and proactive awareness campaigns, which have previously been successful, but should be co-developed with service users and tested on social media.

## Supplementary Material

Supplemental Material

## Data Availability

The data that support the findings of this study are available from the corresponding author, S.J., upon reasonable request.
