# Supplementary material for: Investigating mental health service user views of stigma on Twitter during COVID-19: a mixed-methods study
Source: J Ment Health. 2022 Jul 3;31(4):576–84. doi: 10.1080/09638237.2022.2091763 (PMC9612929; doi:10.1080/09638237.2022.2091763)
Supplement: Supplemental Material [file IJMH_A_2091763_SM5291.docx]

# **Supplementary Material**

# **Methods**

*Procedure*

# Keywords used for tweet extraction

'mental health', 'depression', 'anxiety', 'depres', 'psycho', 'schiz', 'isolat', 'autism', 'autistic', 'Asperger', 'Asperger's' 'ASD', 'anorexia', 'anorexic', 'bulimia', 'bulimic', 'OSFED', 'EDNOS', 'OCD', 'obsessive compulsive disorder', ‘addict’, ‘drug’, ‘alcohol’.

# Topic guide

[Introduce yourself and study topic – refer to information sheet]

- What were you thinking when you were classifying tweets into either stigmatising or not?
  - How did you define stigma? For instance, did you base it on how the tweet may you feel?
- What do you think are the biggest issues with reading/finding these negative tweets online?
- How has the COVID-19 pandemic affected your social media use?
- How has social media affected you during the COVID-19 pandemic? Do you feel better/worse because of social media?
- How has social media content changed since January to now – have you noticed differences in the nature of content from January to now?
- How do you feel about your (public) social media data used in mental health research?
  - Would you like to be consented for your social media to be used? If so, how? E.g., via a direct message on twitter?

**Results**

| **Supplementary Table 1.** Breakdown of number of stigmatising and non-stigmatising tweets by condition, as rated by mental health service users. | | | | |
| --- | --- | --- | --- | --- |
| **Condition** | **Stigmatising, n** | **Not stigmatising, n** | **Total tweets, n** | **Percentage stigmatising (%)** |
| Schizophrenia | 411 | 123 | 534 | 76.97% |
| Eating Disorder | 179 | 185 | 364 | 49.18% |
| OCD | 222 | 312 | 534 | 41.57% |
| Addiction | 30 | 70 | 100 | 30.00% |
| Autism | 120 | 414 | 534 | 22.47% |
| Depression/Anxiety | 139 | 495 | 634 | 21.92% |
| **Total** | **1101** | **1599** | **2700** | **40.78%** |

| **Supplementary Table 2.** Participant characteristics within each focus group. | | | | | | |
| --- | --- | --- | --- | --- | --- | --- |
| **Characteristic** | | **Group 1  (n = 5)** | **Group 2  (n = 5)** | **Group 3  (n = 8)** | **Group 4  (n = 5)** | **Total (N = 23)** |
| **Gender (n, %)** | |  |  |  |  |  |
|  | Female | 4 (80%) | 3 (60%) | 5 (62.5%) | 5 (100%) | 17 (74%) |
|  | Male | 1 (20%) | 1 (20%) | 2 (25%) | – | 4 (17.3%) |
|  | Other | – | 1 (20%) | 1 (12.5%) | – | 2 (8.7%) |
| **Age (years), mean (SD) Range** | | 49.6 (9.0) (37 - 62) | 41.2 (19.4) (19 - 60) | 45 (17.3) (19 - 69) | 46.2 (15.8) (28 - 60) | 45.4 (15.2)  (19 - 69) |
| **Ethnicity (n, %)** | |  |  |  |  |  |
|  | Asian | – | 2 (40%) | 1 (12.5%) | – | 3 (13%) |
|  | Black African | – | – | 1 (12.5%) | – | 1 (4.25%) |
|  | White | 5 (100%) | 3 (60%) | 6 (75%) | 5 (100%) | 19 (82.6%) |
| **Highest Qualification (n, %)** | |  |  |  |  |  |
|  | No qualifications | – | 1 (20%) | – | – | 1 (4.3%) |
|  | A-Level or equivalent | – | 2 (40%) | 4 (50%) | 1 (20%) | 7 (30.4%) |
|  | Degree level or above | 5 (100%) | 2 (40%) | 4 (50%) | 4 (80%) | 15 (65.2%) |
| **Age completed studies (years), mean (SD) Range** | | 31.4 (8.9) (23 - 45) | 19.8 (2.7) (16 - 22) | 20.3 (2.8) (16 - 25) | 21.4 (2.7)  (17 - 24) | 22.8 (6.4) (16 - 45) |
| **Currently receiving mental health support (n, %)** | | 4 (80%) | 3 (60%) | 6 (75%) | 3 (60%) | 16 (70%) |
| **Member checking group attendance  (n, %)** | | 5 (100%) | 3 (60%) | 8 (100%) | 5 (100%) | 21 (91%) |
